# Assessing policy readiness for climate-informed infectious disease early warning systems in Vietnam: a scoping review and stakeholder consultation

**DOI:** 10.1080/16549716.2026.2757958

**Published:** 2026-11-03

**Authors:** Cong Tuan Pham, Ha Thu Nguyen, Hong H.T.C Le, Vinh Bui, Hai Phung, Dung Phung, Cordia Chu

**Affiliations:** a Centre for Environment and Population Health, Griffith University, Brisbane, Queensland, Australia; b School of Preventive Medicine and Public Health, Hanoi Medical University, Hanoi, Vietnam; c School of Public Health, The University of Queensland, Brisbane, Queensland, Australia; d Faculty of Science and Engineering, Southern Cross University, Lismore, New South Wales, Australia; e School of Medicine and Dentistry, Griffith University, Gold Coast, Queensland, Australia

**Keywords:** Health policy, climate-informed early warning systems, infectious disease, climate change and health, governance

## Abstract

**Background:**

Climate change is reshaping infectious disease patterns, increasing the need for climate-informed early warning systems (EWSs). However, the extent to which Vietnam’s governance frameworks support their development and implementation remains unclear.

**Objectives:**

To assess policies and guidelines governing infectious disease EWSs in Vietnam and identify gaps and opportunities to strengthen system readiness.

**Methods:**

A policy review using a modified scoping review approach was supplemented by qualitative stakeholder consultations. National regulatory documents issued since 2000 and in effect as of July 2026 were identified through Vietnamese legal databases. Documents were analysed thematically and mapped against the core components of EWSs. Twelve key stakeholders were interviewed to contextualise policy findings and explore implementation challenges.

**Results:**

Fifteen policy documents met the inclusion criteria. Vietnam has established strong legal and policy foundations for disease reporting, outbreak investigation, emergency response, and intersectoral coordination. Recent policies increasingly recognise climate change adaptation, environmental risks, digital transformation, and cross-sector collaboration, creating an enabling environment for the development of EWSs. However, these policies primarily provide strategic direction and institutional support rather than operational guidance for implementing EWSs. Key gaps include fragmented governance arrangements for data sharing and integration across meteorological, epidemiological, vector, laboratory, and vulnerability datasets; limited guidance on model validation and adoption; weak mechanisms linking forecasts to authorised anticipatory actions; and constraints related to financing and workforce capacity.

**Conclusions:**

Strengthening policies and operational guidance for forecasting, data integration, and forecast-based action will be critical to enhancing preparedness for and responses to climate-sensitive infectious diseases in Vietnam.

## Paper Context

- **Main findings**: Vietnam has strong policy foundations for climate-informed infectious disease EWSs, but gaps remain in climate-health integration, forecast-based action, data sharing, and sustainable implementation.
- **Added knowledge**: This study shows that the main barriers to climate-informed EWSs are governance and institutional challenges rather than technical limitations.
- **Global health impact for policy and action**: Strengthening climate-health data integration, warning-to-action mechanisms, and anticipatory financing can support more proactive and climate-resilient disease prevention.

## Background

Climate variability and climate change are increasingly recognised as major drivers of infectious disease dynamics and transmission patterns [1]. Advances in risk assessment methods, data collection, surveillance systems, and modelling technologies have created new opportunities for the development and implementation of climate-informed early warning systems (EWSs) that can anticipate disease outbreaks and facilitate timely, effective public health action [2,3]. An infectious disease EWS is a system that uses climate, meteorological, hydrological, environmental, epidemiological, and vulnerability data to assess and forecast disease risk; converts risk information into warnings; communicates these warnings to authorised actors and at-risk populations; and links warnings to predefined preparedness or response actions [2,3]. Effective EWSs depend on coherent policy support across all functional domains [4–7]. Policy frameworks support risk knowledge in EWSs by mandating the systematic collection, analysis, and mapping of hazard, exposure, and vulnerability data [8,9]. Monitoring and warning systems rely on continuous data collection and scientific forecasting to detect emerging threats [4–7]. Effective communication ensures that warnings are timely, actionable, and accessible to at-risk populations, with trusted authorities playing a key role in enhancing credibility and uptake [10]. Finally, policy frameworks support EWS response capacity through clear agency roles, interoperable data sharing, preparedness planning, community awareness, and local preparedness [4–7,10].

As a country highly vulnerable to climate change, Vietnam has increasingly integrated climate-informed approaches into health and adaptation policies [11,12]. However, there remains limited understanding of the extent to which existing policies and guidelines in Vietnam support the development and implementation of EWSs for infectious diseases. This study addresses this knowledge gap by conducting a scoping review of policies and guidelines relevant to climate-informed infectious disease early warning and response in Vietnam. Specifically, the review aims to map governance instruments against the core components of an EWS and assess the extent to which the current policy environment enables the operationalisation of infectious disease EWSs.

## Methods

The policy review employed a modified scoping review approach, informed by the Joanna Briggs Institute (JBI) methodology for scoping reviews [13], and reported in accordance with the PRISMA-ScR guideline [14], supplemented by qualitative stakeholder consultation. The review was registered with the Open Science Framework (14 August 2025; https://doi.org/10.17605/OSF.IO/RZ7PS).

## Policy review

### Information sources and search strategy

Policy and regulatory documents were identified from Vietnamese regulatory databases and Vietnamese government official portals, including Thư Viện Pháp Luật, the National Legal Portal, the Vietnam Ministry of Health, the Ministry of Agriculture and Environment, and the Vietnam Government Portal. Structured search strategies were developed in Vietnamese using combinations of keywords related to ‘early warning systems/early warning/prediction’, ‘infectious diseases/disease outbreak’, and ‘climate/weather’. Detailed document search strategies are presented in Supplementary Table 1. Additional policy documents were identified through recommendations from subject-matter experts.

### Eligibility criteria and selection process

Documents were considered eligible for inclusion if they were policies or guidelines related to EWSs or climate-sensitive infectious disease surveillance and were written in Vietnamese. Eligibility was further restricted to documents published from 2000 onward that remained in effect as of 10 July 2026. Documents were excluded if they had been superseded by updated versions or if they focused exclusively on hazard warning systems unrelated to health.

All identified documents were compiled into a Microsoft Excel database, and duplicates were removed. Two independent reviewers (CTP and HTN) conducted a two-stage screening process. First, titles and scope descriptions were assessed for eligibility. Second, full-text screening was undertaken for documents meeting the inclusion criteria. Discrepancies were resolved through discussion until consensus was reached.

### Data extraction

Data extraction was conducted by the first author using a standardised extraction template and independently reviewed by a second researcher. Coding against the EWS framework was reviewed by the research team, and discrepancies were resolved through discussion and consensus. Data were extracted using a standardised charting form that recorded the document title, issuing agency, and year of enactment. The form also captured policy objectives; references to climate and environmental data; linkages with epidemiological surveillance systems; and policy provisions and guidance related to risk knowledge and assessment, detection, monitoring and forecasting, early warning dissemination and communication, and preparedness and response to climate-sensitive infectious diseases. In addition, information was collected on intersectoral coordination mechanisms, funding arrangements, and workforce capacity and training components relevant to EWSs.

## Stakeholder consultation

To enhance validity and contextual relevance, preliminary policy review findings were refined through stakeholder consultation. Policy review findings informed purposive sampling for the qualitative phase to capture diverse perspectives across governance levels and areas of expertise. Participants were purposively selected based on their involvement in the development, implementation, or oversight of policies and activities relevant to climate-sensitive infectious disease EWSs in Vietnam. The National Institute of Hygiene and Epidemiology (NIHE) invited relevant organisations to nominate staff with appropriate expertise, and all 12 invited participants agreed to take part (participant characteristics are presented in Supplementary Table 2). Provincial participants were recruited from two Mekong Delta provinces to provide perspectives from one of Vietnam’s regions most vulnerable to climate change and climate-sensitive infectious diseases. Given the focused scope of the study and the specialised nature of the stakeholder population, the sample provided sufficient information power to address the study objectives, as participants occupied key roles across sectors and administrative levels relevant to infectious disease EWS governance.

Interviews lasted approximately 45 minutes and explored participants’ perspectives on the interpretation of policy findings, implementation challenges, governance gaps, and recommendations for strengthening EWSs. Of the twelve interviews conducted, nine were undertaken in person at participants’ workplaces, and three were conducted virtually. Interviews were audio-recorded, supplemented with field notes.

## Data analysis

This study applied a hybrid thematic analysis integrating deductive and inductive coding approaches. Policy documents were first deductively mapped against the four EWS components: (1) risk knowledge and assessment, (2) detection, monitoring and forecasting, (3) warning dissemination and communication, and (4) preparedness and response capacity. Policies were systematically mapped against these domains to identify strengths, overlaps, inconsistencies, and governance gaps. Policy readiness was assessed by examining the extent to which documents provided legal mandates, governance arrangements, operational guidance, implementation mechanisms, and resource commitments relevant to each EWS component (assessment criteria and illustrative examples are presented in Supplementary Table 4). The findings informed recommendations to support integrated, scalable, and sustainable EWSs in Vietnam.

Interview transcripts were then analysed inductively to identify perceived implementation barriers and governance gaps [15,16]. Verbatim interview transcripts were imported into NVivo 14 for qualitative analysis. Initial line-by-line open coding was conducted by the first author to identify meaningful concepts and patterns. Related codes were subsequently grouped into broader categories through iterative coding and constant comparison. Emerging codes, categories, and interpretations were regularly reviewed by the research team, and discrepancies were discussed until consensus was reached.

Finally, findings from both components were triangulated to identify areas of convergence, divergence, and complementarity between policy provisions and stakeholder perspectives. Integrated themes were subsequently developed by synthesising evidence across the policy review and stakeholder consultations. The coding framework was progressively refined through regular discussions among the research team until consensus was reached on the final themes.

All interviews were conducted in Vietnamese, audio-recorded, and transcribed verbatim. Coding and analysis were undertaken using the original Vietnamese transcripts to preserve contextual meaning and minimise interpretive loss. Quotations included in the manuscript were translated into English by bilingual researchers and independently reviewed by a second team member to ensure conceptual equivalence and consistency in the interpretation of key governance concepts.

## Credibility, trustworthiness, and researcher reflexivity

Several strategies were employed to enhance the credibility and trustworthiness of the findings. Methodological triangulation was achieved by integrating evidence from policy documents and interviews with stakeholders across multiple governance levels and stakeholder groups, allowing findings to be corroborated across data sources and perspectives. The analysis followed an iterative and collaborative process. Initial coding was conducted by the first author, with the coding framework and developing interpretations regularly reviewed by the research team. An audit trail, including analytical memos, coding decisions, and meeting records, was maintained to enhance transparency and dependability.

The policy review and interviews were conducted by trained public health researchers (CTP, HTN, and HHTCL), who were also involved in data analysis, enabling iterative reflection between data collection and interpretation. Although the interviewers had indirect professional connections with participants through public health networks, they had no direct supervisory or implementation relationships. Participants were informed of the researchers’ academic roles and study objectives before the interviews. Reflexive notes were maintained throughout data collection and analysis and were regularly discussed within the research team to ensure interpretations remained grounded in the data.

## Results

The search yielded 235 records after duplicate removal. Following title and full-text screening, 15 policy and regulatory documents met the inclusion criteria (Figure 1).

**Figure 1.**
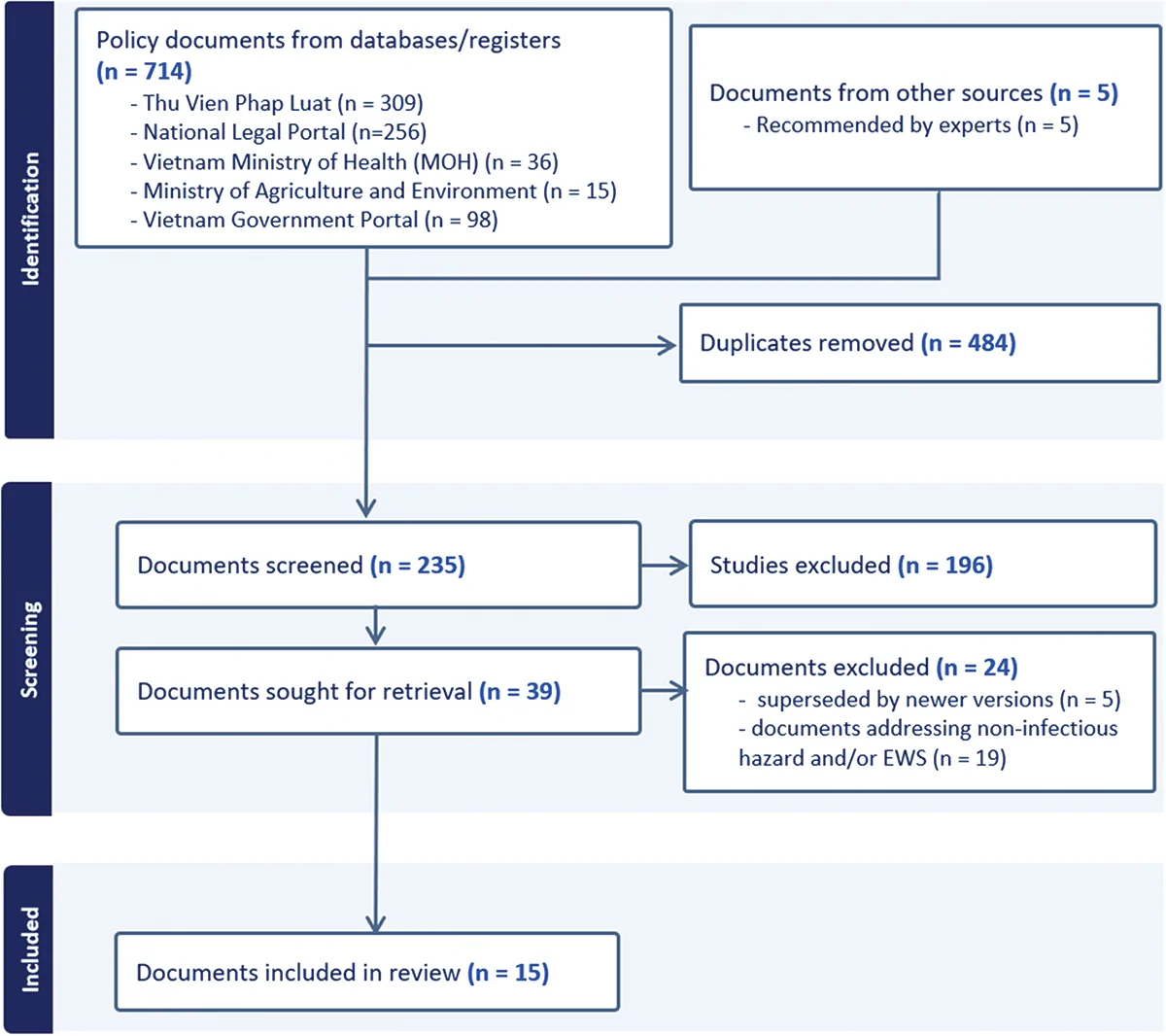
PRISMA flowchart of policy document selection.

The selected documents, issued between 2015 and 2026, comprised laws (*n* = 3), decrees and national resolutions (*n* = 4), and ministerial circulars and decisions (*n* = 8). More than half of these documents (*n* = 8) were introduced within the past two and a half years (2024–2026), indicating increasing policy attention to EWS for infectious diseases. Findings were interpreted with consideration of the differing legal authority and enforceability of policy instruments. While laws, decrees, and circulars establish legally binding mandates and implementation requirements, several provisions related to climate-informed EWSs were contained within strategic plans and technical decisions that primarily provide policy direction and operational guidance. Key policy and regulatory instruments relevant to infectious disease EWSs in Vietnam and their relevance to core EWS functions are summarised in Table 1.

**Table 1.** Policy and regulatory documents relevant to infectious disease EWS in Vietnam.

No.	Policy / Guideline	Year	Issuing Body	Type	Focus	Relevance to EWS
1	Law No. 90/2015/QH13 on Hydrometeorology	2015	National Assembly	Law	Management and use of climate and hydrological data	Provides essential climate data inputs for EWSs; not linked to health operations
2	Decision 4283/QĐ-BYT	2016	Ministry of Health	Technical decision	Standardised case definitions	Supports detection of climate-sensitive diseases; no predictive capacity
3	Decree 89/2018/NĐ-CP	2018	Government	Decree	Border health quarantine and surveillance	Supports early detection at borders; no climate or forecasting component
4	Decision 2018/QĐ-BYT	2018	Ministry of Health	Technical decision	Event-based surveillance (EBS)	Expands early detection using multiple data sources; no climate integration
5	Decision 3929/QĐ-BYT	2018	Ministry of Health	Strategic plan	SDGs implementation, climate adaptation, environmental health	Links climate change and health; calls for EWSs
6	Circular 06/2023/TT-BTNMT	2023	Ministry of Natural Resources and Environment	Circular	Guidelines for integrating climate change response content into strategies and plans.	Indirectly supports EWSs by enabling the cross-sectoral data sharing, risk assessment protocols, and strategic planning required to integrate a disease EWSs to the health system.
7	Law No. 18/2023/QH15 on Civil Defense	2023	National Assembly	Law	Rules and operations of civil defence	Provisions for EWSs and intersectoral coordination for climate-related epidemics and disaster response.
8	Decision 1089/QĐ-BYT	2024	Ministry of Health	Strategic plan	Scientific research and innovation	Supports development of disease forecasting (HFMD)
9	Decision 3990/QĐ-BYT	2024	Ministry of Health	Strategic plan	Green growth and climate adaptation in health	Strengthens environmental health and climate adaptation capacity
10	Law No. 114/2025/QH15 on Disease Prevention	2025	National Assembly	Law	Comprehensive prevention framework, digital health, forecasting	Enables forecasting and early warning; lacks operational climate guidance
11	Resolution 282/NQ-CP	2025	Government	Resolution	Health system strengthening, digital transformation	Promotes AI, big data, early warning; climate integration remains general
12	Resolution 147/NQ-CP	2025	Government	Resolution	Non-traditional security threats (including epidemics, climate risks)	Supports forecasting, early warning, and multi-sectoral response
13	Decree 165/2025/NĐ-CP	2025	Government	Decree	Data governance, sharing, interoperability	Critical for integrated data systems and cross-sector EWSs functionality
14	Decision 631/QĐ-BYT	2026	Ministry of Health	Operational plan	Annual infectious disease control priorities	Recognises climate risks but lacks defined forecasting thresholds
15	Circular 15/2026/TT-BYT	2026	Ministry of Health	Circular	Regulations detailing certain provisions of the Law on Disease Prevention	Represents the most explicit EWS-related document; including climate variables and warning threshold at the commune level. Warning content includes description of risk by time, location, scale, and scope.

The reviewed policies demonstrate a clear progression from a conventional communicable disease control framework toward a broader, prevention-oriented, climate-aware, and risk-based governance model. Six key themes (presented in Table 2) were developed through integration of the deductive policy mapping and inductive stakeholder analysis (see the analytical integration matrix in Supplementary Table 3).

**Table 2.** Key themes for reviewing climate-informed infectious disease EWS policies in Vietnam.

Theme	Subthemes
Theme 1: Vietnam’s policy framework has expanded beyond pathogen-centred risk but has not yet operationalised climate-informed risk assessment	Growing Recognition of Climate, Environmental, and Social Determinants of Risk
	Limited Operationalisation of Climate-Informed Risk Assessment
Theme 2: Advanced epidemiological surveillance but limited operational integration of climate information for anticipatory warning	Established surveillance and reporting infrastructure
	Emerging foundations for integrated and digital surveillance
	Persistent barriers to climate-informed early warning
Theme 3: Comprehensive reporting infrastructure but limited operational capacity for anticipatory warning and action	Well-established institutional arrangements for disease reporting and risk communication
	Weak links between warning dissemination and anticipatory action
Theme 4: Preparedness and response capacities are robust, but remain largely reactive rather than forecast-based	Comprehensive preparedness and outbreak response capacity
	Reactive rather than anticipatory response mechanisms
	Limited institutional legitimacy for forecast-based decision-making
Theme 5: Intersectoral coordination is widely mandated, but data-sharing governance remains fragmented	Intersectoral coordination as a policy principle
	Limited data-sharing and interoperability mechanisms
Theme 6: Resource and capacity constraints threaten sustainable EWS implementation	Resource and financing constraints for EWS implementation
	Workforce and local implementation capacity gaps

## Climate-informed risk assessment remains underdeveloped

### Growing recognition of climate, environmental, and social determinants of risk

Vietnam’s infectious disease policy framework is comparatively well developed in relation to risk knowledge, especially through provisions for pathogen classification, disease surveillance systems, and the monitoring of epidemiological exposure. Earlier instruments, such as Decision 4283/QĐ-BYT (2016), emphasised surveillance and outbreak control, with limited attention to population vulnerability, differential exposure, or broader determinants of health risk. More recent policy instruments indicate a gradual broadening of how infectious disease risk is understood. Resolution 147/NQ-CP (2025), Decision 3929/QĐ-BYT (2018), Resolution 282/NQ-CP (2025), and the Law on Disease Prevention (2025) incorporate considerations of vulnerable populations, geographic inequalities, environmental conditions, and climate-related threats. This reflects a shift from a predominantly pathogen-centred perspective towards a more comprehensive public health understanding of risk.

Recognition of the links between climate, environmental change, and infectious disease transmission has become increasingly evident across the policy framework. Circular 15/2026/TT-BYT (2026), replacing Circulars 17/2019/TT-BYT and 54/2015/TT-BYT, explicitly incorporates climatic variables such as temperature, rainfall, humidity, and seasonality into infectious disease surveillance activities. Similarly, Decision 631/QĐ-BYT (2026) identifies climate change, natural disasters, urbanisation, and population mobility as key drivers of disease emergence and transmission.

Stakeholder consultations further confirmed that the recognition of the climatic and environmental determinants of infectious disease risk extends beyond policy documents and is increasingly reflected in expert and policymaker perspectives. Participants explicitly recognised the influence of climate variability and climate change on the spatial and temporal dynamics of climate-sensitive diseases, particularly dengue. As one policymaker noted:

The increase [in dengue cases] depends on weather and climate conditions … The warming trend associated with climate change is very clear … the number of cases in northern provinces is increasing more rapidly. (Policymaker 01)

The convergence between policy provisions and stakeholder perspectives suggests a growing consensus regarding the importance of climate and environmental factors in shaping infectious disease risks in Vietnam.

Beyond the health sector, Circular 06/2023/TT-BTNMT (2023) provides guidance for integrating climate change adaptation into sectoral planning and development strategies. While not directly focused on health, this policy creates an enabling environment for intersectoral collaboration and supports the incorporation of climate considerations into health planning and risk assessment processes.

### Limited operationalisation of climate-informed risk assessment

Despite these advances, important gaps remain in translating climate-sensitive risk concepts into operational practice. Although several policy documents recognise seasonal patterns, temperature, rainfall, humidity, floods, storms, environmental pollution, and climate change as factors influencing infectious disease risks, few policies specify how these variables should be systematically incorporated into surveillance, risk assessment, or decision-making processes. None of the reviewed policy documents explicitly establish mechanisms for integrating meteorological and epidemiological datasets, define institutional responsibilities for disease risk mapping, or provide methodologies for assessing climate-related health vulnerabilities.

Stakeholders also highlighted the limited integration of climate, environmental, and non-health data into infectious disease risk assessment. One policymaker explained:

At present, disease risk assessment is mainly based on case data. Other data such as vector, laboratory, and hydro-meteorological data are very limited and cannot be updated in real time. (Policymaker 02)

Consequently, disease forecasting and risk assessment continue to rely predominantly on reported case data, while the systematic incorporation of climate-sensitive vulnerability indicators and non-health data remains limited.

## Advanced epidemiological surveillance system with limited climate integration

### Established surveillance and reporting infrastructure

Detection and monitoring represent the strongest component of Vietnam’s infectious disease policy framework. Circular 15/2026/TT-BYT and the Law on Disease Prevention (2025) provide a framework for infectious disease surveillance, reporting, and outbreak warning, with clearly defined reporting mechanisms, warning thresholds, and responsibilities across administrative levels.

Stakeholders similarly viewed Vietnam’s surveillance infrastructure as relatively well developed, particularly through the national electronic infectious disease reporting system established under Circular 54/2015/TT-BYT and subsequently replaced by Circular 15/2026/TT-BYT (2026). This platform enables rapid reporting of infectious disease information from local health facilities to national authorities.

As one policymaker explained:

We currently have the infectious disease reporting system under Circular 54 … The Department of Disease Prevention can receive reports from all units through the software immediately after they are submitted. (Policymaker 01)

This digital reporting infrastructure provides an important foundation for the development of EWSs by facilitating timely data collection and information flow across the health system.

However, stakeholders consistently noted that the current surveillance system primarily functions as a case-reporting and outbreak-monitoring mechanism. Its principal strength lies in detecting and reporting cases after they occur rather than anticipating outbreaks before transmission intensifies. As one policymaker observed:

Current surveillance is mainly based on cases from treatment facilities, which often involves a delay and therefore loses the predictive value. (Policymaker 03)

Reliance on facility-based reporting means that surveillance information is generated only after individuals seek care, limiting opportunities for earlier preventive action. While essential for outbreak detection and response, these data alone are insufficient to support anticipatory warning unless combined with upstream indicators of disease risk.

### Emerging foundations for integrated and digital surveillance

Recent policies have strengthened the enabling environment for more integrated surveillance approaches. Decision 2018/QĐ-BYT (2018) introduced event-based surveillance (EBS), allowing signals to be identified from multiple sources, including communities, laboratories, healthcare facilities, and media reports. Circular 15/2026/TT-BYT (2026) subsequently strengthened the integration of indicator-based and EBS with outbreak investigation, risk assessment, and response procedures. These developments reflect a growing recognition of the need for more flexible and timely surveillance systems capable of identifying emerging public health threats beyond routine case reporting.

Furthermore, existing policies establish important institutional foundations for cross-sectoral data integration. Decree 165/2025/NĐ-CP provides provisions for data governance, interoperability, and information sharing, while the Law on Meteorology and Hydrology (2015) establishes a framework for the collection, management, and dissemination of meteorological information.

Stakeholders recognised the potential value of incorporating a broader range of epidemiological, environmental, and social indicators into infectious disease forecasting. One policymaker described possible inputs for future predictive models:

For example, we may include input data such as case numbers, deaths, hospitalisations, temperature, population, climate, weather, and other factors such as people’s practices. (Policymaker 01)

This suggests a growing acceptance of meteorological and contextual variables as relevant components of disease prediction. Together, these policies create enabling conditions for integrating climate and health data, providing a foundation for the development of climate-informed infectious disease surveillance and EWSs.

### Persistent barriers to climate-informed early warning

Despite these advances, Vietnam’s policy framework remains predominantly oriented towards disease detection and response rather than climate-informed anticipatory warning. Although several recent policies, including Resolution 282/NQ-CP (2025), Decision 631/QĐ-BYT (2026), Circular 15/2026/TT-BYT, and the Law on Disease Prevention (2025), reference forecasting, artificial intelligence, and big data applications, they provide limited guidance on forecasting methodologies, modelling standards, institutional responsibilities, or operational procedures for predictive analysis.

Stakeholders reported that climate-informed early warning initiatives, such as D-MOSS, remain largely pilot or project-based rather than institutionalised components of the national surveillance system. As one policymaker explained:

At present, there is no official model applied nationwide; existing models remain at the level of projects or pilots. (Policymaker 01)

A major challenge identified by participants was the weak integration of health and meteorological information systems. Although relevant climate and weather data are available, they are often maintained outside the health sector and are not routinely connected to disease surveillance platforms. The primary challenge is not the absence of climate information but the lack of interoperable systems and automated data-sharing mechanisms capable of supporting timely, scalable, and reliable forecasting.

A leader of a meteorological research centre involved in developing a dengue EWS explained:

Weather data, for example data from observation stations, are protected, so they are not easy to access. (Policymaker 04)

Restricted access to climate data and fragmented governance arrangements may hinder the development and routine use of climate-informed surveillance and EWSs. Without formal data-sharing mechanisms across meteorological, health, research, and technology sectors, climate-informed approaches are likely to remain project-based rather than institutionalised. Effective implementation requires accessible, interoperable, quality-assured, and routinely updated datasets embedded within decision-making processes.

## Comprehensive reporting systems but underdeveloped warning architecture

### Well-established institutional arrangements for disease reporting and risk communication

Vietnam’s policy environment establishes a comprehensive infectious disease reporting system, with clear provisions for reporting responsibilities, notifiable events, reporting timelines, and electronic reporting procedures. Together, these arrangements create structured information flows across administrative levels and provide a robust foundation for disease surveillance, notification, and information exchange. Supported by digital reporting systems and defined governance mechanisms, the framework demonstrates strong institutional capacity for collecting and transmitting surveillance data.

Several policy documents, including Circular 15/2026/TT-BYT (2026), Decision 2018/QĐ-BYT (2018), and Decision 3929/QĐ-BYT (2018), emphasise communication with the public through mass media, social media, community-based channels, and health education activities. Recent policies also highlight the importance of proactive information sharing and behavioural change communication as part of infectious disease prevention and control. These provisions indicate a growing recognition of public communication as an essential component of outbreak management.

### Weak links between warning dissemination and anticipatory action

While policies encourage dissemination of health information, they provide limited guidance on how surveillance findings, forecast information, or emerging risks should be translated into structured warnings for different audiences. In particular, the reviewed documents do not establish standardised warning levels, alert categories, risk classifications, or dissemination protocols that connect risk assessments with specific warning messages. Consequently, communication mechanisms exist, but their role within a broader early warning architecture remains insufficiently defined.

Furthermore, stakeholders highlighted that the effectiveness of an EWS depends not only on generating information but also on ensuring that warnings trigger timely and appropriate action. Although Vietnam has experience with EBS and the identification of early signals, participants noted that these signals do not always result in operational responses. One technical officer explained:

EBS can be one or two days earlier. Those one or two days are valuable, but most of the time we do not act strongly on that. (Technical Officer 02)

This suggests that the benefits of earlier detection may not be fully realised when warning signals are not embedded within formal decision-making processes. Early information alone is insufficient if responsibilities, response thresholds, and procedures for action are not clearly defined. The findings indicate that the key challenge extends beyond communication itself; rather, it concerns the governance arrangements that determine how warnings acquire authority, legitimacy, and practical consequences. Without predefined alert levels, authorised warning protocols, and corresponding response measures, surveillance information may remain informative rather than operationally actionable.

## Robust response architecture but limited anticipatory action

### Comprehensive preparedness and outbreak response capacity

Vietnam’s infectious disease governance framework includes a well-developed legal and operational architecture for outbreak preparedness and response. The Law on Disease Prevention (2025) establishes a comprehensive framework for epidemic management. Operational preparedness is further supported by Decree 89/2018/NĐ-CP and Circular 15/2026/TT-BYT, which provide guidance on border health measures and threshold-based response mechanisms. The reviewed policies collectively provide a broad and coherent system for epidemic control, covering epidemic declaration, steering committee activation, isolation and treatment, environmental sanitation, disinfection, vaccination campaigns, vector control, movement restrictions, resource mobilisation, and emergency operations. These provisions incorporate active and EBS, border health control, genomic surveillance, laboratory biosafety, rapid response teams, emergency operations centres, stockpiles, logistics, treatment readiness, and risk communication. Decision 631/QĐ-BYT (2026) complements these provisions by defining disease control targets and interventions.

Together, these findings indicate that preparedness and response constitute one of the most mature components of Vietnam’s infectious disease governance system.

### Reactive rather than anticipatory response mechanisms

Despite well-developed preparedness and response capacity, the current framework remains predominantly reactive. Most interventions are activated after cases, clusters, outbreaks, elevated vector indices, or other epidemiological signals have been detected. While policies establish clear procedures for responding to realised risks, they provide limited guidance on acting upon forecast risks before transmission accelerates.

Stakeholder accounts reinforced this distinction. At the local level, health workers appeared more accustomed to responding to outbreaks than initiating preventive interventions based on projected risk. One practitioner explained:

At the commune health station, we only respond to outbreaks … We usually do not initiate prevention campaigns ourselves. (Practitioner 02)

Disease preventive action is constrained not only by available information but also by administrative norms governing who is authorised to initiate interventions. Commune health facilities may be reluctant to undertake pre-emptive measures without higher-level direction or formal outbreak signals.

Several participants similarly noted that actions are often triggered only after epidemiological or entomological indicators exceed established thresholds. As one policymaker observed:

If we only act when vector indices are already high, then it is already reactive. (Policymaker 02)

From a climate-informed early warning perspective, response systems should enable action before conventional surveillance indicators reach critical levels. However, current policies do not clearly define such forecast-based action pathways. Consequently, Vietnam’s response system appears well equipped to manage outbreaks once risks have materialised but less developed in supporting preventive action based on anticipated risks.

### Limited institutional legitimacy for forecast-based decision-making

A further challenge identified by stakeholders was the lack of legal and administrative mechanisms for acting on predictive information. Although model outputs and forecasting tools were generally viewed as potentially useful for planning purposes, participants questioned whether they could legitimately trigger official action, resource allocation, or public health interventions. As one technical officer explained:

… if you rely on software, and we do not yet know how accurate that software is, it may be a basis for proposing a plan, but it has no legal basis. (Policymaker 03)

Similarly, local stakeholders emphasised that warning information requires formal authorisation before it can be translated into action. One practitioner described the challenge:

They ask: which official document regulates this? (Practitioner 02)

These accounts suggest that forecast information does not automatically acquire operational authority simply because it is technically available. Local governments and health facilities may require recognised legal mandates before allocating resources, mobilising personnel, or implementing preventive measures. Warning dissemination is therefore not solely a technical issue but also one of administrative legitimacy and institutional governance.

Participants also highlighted the absence of formal procedures for evaluating and operationalising predictive models. As one policymaker explained:

There is currently no regulation on this … Whether a model is selected or not would require a specific process and guidance. (Policymaker 01)

This reveals a significant gap between strategic support for innovation and operational implementation. Current policies do not clearly specify how predictive models should be selected, validated, approved, maintained, or incorporated into decision-making processes. Nor do they provide guidance on how forecast uncertainty, confidence levels, or false alarms should be interpreted when making public health decisions.

In addition, stakeholders expressed differing views on the adoption of forecasting models. While some viewed climate-informed forecasting as an opportunity to support proactive disease prevention and resource allocation, others raised concerns about forecast uncertainty, accuracy, and institutional accountability. These differences highlight the need for stronger validation frameworks and operational guidance before forecasts can routinely inform public health action.

Beyond implementation challenges, stakeholders highlighted concerns regarding the long-term sustainability of EWSs. Several participants argued that warning systems are unlikely to become operational unless they are formally embedded within existing governance and programme structures.

As one practitioner stated:

There needs to be an official guideline, and it should be incorporated into the routine health programmes itself … A document alone is not enough. (Practitioner 02)

This suggests that EWSs require more than regulatory recognition. To influence routine practice, they may need to be incorporated into funded programmes, the annual implementation plans, technical guidelines, performance frameworks, and institutional mandates. Without such integration, EWSs risk remaining external tools or pilot innovations rather than becoming routine components of public health practice.

## Mandated coordination but fragmented data-sharing architecture

### Intersectoral coordination as a policy principle

Vietnam’s infectious disease governance framework consistently recognises the importance of intersectoral coordination for disease prevention and control. This is particularly relevant for climate-sensitive infectious diseases, where transmission is influenced not only by epidemiological factors but also by climate variability, hydrological conditions, vector ecology, environmental management, animal health, population mobility, sanitation, and disaster-related risks.

Across the reviewed policy documents, collaboration is emphasised in relation to outbreak response, border health control, zoonotic disease prevention, vector control, environmental sanitation, school-based prevention programmes, and emergency mobilisation. Several policies establish coordination mechanisms between the health sector and a range of non-health actors, including education authorities, agricultural agencies, customs, border management authorities, police, military units, port authorities, animal and plant quarantine services, and local governments.

Recent policy directions further strengthen this multisectoral approach through references to One Health, global health security, and cross-ministerial collaboration. Collectively, these provisions establish a clear institutional mandate for collaborative infectious disease prevention and control and provide an important foundation for EWSs.

### Limited data-sharing and interoperability mechanisms

Despite policy emphasis on coordination, the reviewed documents provide comparatively limited guidance on how information should be exchanged across sectors in practice. Mechanisms for routine, real-time, and interoperable data sharing between health and meteorological agencies remain weakly specified. This represents a significant limitation for infectious disease EWSs, which rely on the integration of diverse datasets, including epidemiological, laboratory, vector, meteorological, hydrological, environmental, animal health, demographic, and vulnerability information.

The operationalisation of EWSs depends not only on technical model development but also on predictable governance arrangements for data access, tool validation, institutional engagement, and responsibility allocation. Where these governance mechanisms are unclear, implementation is likely to remain fragmented and project-based. Although many participants described data-sharing arrangements between sectors as fragmented and constrained by institutional and legal barriers, some stakeholders considered existing mechanisms adequate for current operational needs. These respondents noted that routine disease surveillance and outbreak response activities were already supported through established reporting systems and interagency collaboration, while acknowledging that additional arrangements would be required for climate-informed forecasting.

The policy review similarly found limited guidance regarding routine climate–health data exchange, integration of hydrological information into disease risk assessment, and information sharing between human and animal health systems. Policies rarely specify interoperable data standards, data ownership arrangements, access rights, quality assurance responsibilities, update schedules, or mechanisms for automated and real-time data exchange. Procedures for emergency data sharing during outbreaks, floods, storms, or other climate-related events are also insufficiently defined.

## Resource and capacity constraints

### Resource and financing constraints for EWS implementation

Vietnam’s policy environment demonstrates a growing commitment to preventive health, digital transformation, workforce development, science and technology, artificial intelligence, big data, and health system modernisation. Despite this policy ambition, stakeholders and policy documents highlighted significant resource constraints that may limit implementation. Decision 631/QĐ-BYT identifies several structural challenges affecting preventive medicine and grassroots health systems, including insufficient funding, delays in budget allocation, the absence of a dedicated national target programme for communicable disease control, infrastructure limitations, workforce shortages, and shortages of medicines, supplies, and equipment in some localities.

These constraints have direct implications for EWS implementation. Even where policies support innovation and preventive approaches, local authorities and health facilities may lack the financial, technical, and operational resources required to translate warning information into timely preventive action. Stakeholders also emphasized that existing financing mechanisms are better aligned with routine programmes and outbreak response than with forecast-based action. One practitioner explained:

When we act in advance based on a forecast, there is no plan from higher levels, meaning there is no funding source … we have to request it … . (Practitioner 01)

This perspective was echoed by another participant, who highlighted the outbreak-driven nature of existing financing arrangements:

The principle for funding disease control is that there must already be an outbreak. (Technical Officer 01)

These suggest that financing remains largely reactive and tied to confirmed outbreaks rather than predicted risks. As a result, forecast-based interventions may fall outside established budgeting and approval mechanisms. Because preventive actions often need to be implemented before formal outbreak thresholds are reached, local authorities may face challenges in justifying expenditures or identifying appropriate funding sources. This creates a critical gap between the availability of early warning information and the capacity to act on it. Consequently, even when climate-related disease risks are recognised, the absence of dedicated financing mechanisms for anticipatory action may constrain the practical value of EWSs.

### Workforce and local implementation capacity gaps

Stakeholders identified local implementation capacity as a critical determinant of whether warning information can be translated into action. Infectious disease EWSs require not only forecasting systems at the national level but also local capacity to interpret risk information, communicate warnings, prioritise high-risk areas, and implement preventive interventions.

For climate-sensitive diseases such as dengue, this may involve entomological surveillance, vector control planning, community mobilisation, and environmental management. However, participants reported uneven technical capacity across local health systems, particularly following administrative restructuring. One technical officer noted:

The commune level has not yet been trained in entomological vectors. (Technical Officer 03)

The effectiveness of EWSs depends not only on producing accurate forecasts but also on ensuring that end users possess the technical knowledge and operational authority required to respond appropriately.

Stakeholders also emphasised the importance of ongoing technical maintenance and institutional ownership. A researcher involved in EWS development explained:

There needs to be a technical unit to ensure the maintenance of early warning system on a regular basis … and that relates to funding. (Researcher 02)

Infectious disease EWSs are not one-off technological products. Their long-term operation requires continuous software maintenance, routine data updating, model recalibration, quality assurance, user support, governance oversight, and sustainable financing. While forecasting tools can often be developed through research projects or donor-supported initiatives, their continued effectiveness depends on whether a designated institution is responsible for maintaining and operating them after project funding ends.

The policy framework includes substantial investment in capacity building across surveillance, outbreak investigation, laboratory diagnosis, biosafety, infection prevention and control, vaccination safety, field epidemiology, emergency operations, clinical management, genomic sequencing, simulation exercises, and data management. These provide important foundations for infectious disease preparedness and response. However, capacity development remains focused primarily on conventional epidemiological surveillance and outbreak management. Comparatively less attention is given to competencies required for EWSs, including climate–health data integration, predictive modelling, geospatial analysis, interpretation of probabilistic forecasts, model validation, interoperability, forecast-based decision-making, and risk communication under conditions of uncertainty.

## Discussion

This study provides a systematic and comprehensive assessment and mapping of policies and governance instruments supporting EWS for infectious diseases in Vietnam. A major strength of the current policy framework is that it establishes an enabling foundation for surveillance, reporting, and outbreak response systems, thereby supporting the future development and implementation of EWSs. These strategies demonstrate a sustained, high-level commitment to building resilience against climate-related health risks [17,18].

Despite these advances, the findings suggest that climate-informed risk assessment remains insufficiently operationalised. While policies increasingly recognise the influence of climate variables on infectious disease transmission, disease risk assessment continues to rely predominantly on epidemiological case data, with limited use of meteorological, environmental, vulnerability, or exposure indicators [19].

The analysis also reveals a mismatch between surveillance capacity and anticipatory warning capability. Although Vietnam has demonstrated strong operational capacity for infectious disease surveillance, existing systems primarily support outbreak detection and response after transmission has occurred rather than forecasting future risks. Climate-informed forecasting initiatives remain largely project-based and have not yet been institutionalised within routine public health practice. This gap reflects a broader challenge in many low- and middle-income countries, where surveillance systems are relatively established but predictive analytics and climate-health integration remain underdeveloped [20].

A similar pattern is observed across Southeast Asia, where health systems largely rely on surveillance and case reporting to detect outbreaks after transmission has occurred, while climate-health forecasting remains limited and mostly confined to research or pilot projects [21]. Although countries such as Thailand and Malaysia have developed climate-informed EWS models, these have generally not been integrated into formal warning frameworks or response protocols [21–23]. The absence of operational forecasting limits the ability of the system to shift from early detection to early action [24,25]. Without this capability, opportunities for prevention, particularly for vector-borne diseases, remain underutilized [20].

Despite strong provisions for reporting and communication, policies provide limited guidance on forecast-based triggers, warning protocols, and anticipatory actions, potentially reducing the effectiveness of EWSs. Reporting systems primarily serve internal administrative and surveillance functions, whereas warning systems are designed to communicate risk in a timely and actionable manner to enable preventive action [26]. The absence of standardised warning levels, protocols, and communication triggers limits the effectiveness of the EWS as a comprehensive system [27]. In contrast, other sectors in Vietnam, particularly meteorology and disaster prevention, have established warning frameworks with tiered alert systems and clear communication strategies [5,28]. Adapting similar approaches to infectious disease early warning could enhance clarity, timeliness, and usability of risk information, particularly for subnational authorities and communities.

Preparedness and response capacities were found to be among the strongest components of the policy framework; however, these mechanisms remain predominantly reactive. The absence of forecast-based action protocols, such as pre-emptive vector control, targeted risk communication, or resource mobilisation based on predicted risk, is also reported elsewhere [29,30]. Shifting toward anticipatory action would represent a significant advancement in public health governance [31]. Forecast-based approaches have been increasingly promoted globally as a cost-effective and timely strategy to reduce the impact of climate-sensitive diseases [32]. In Vietnam, dengue represents a suitable entry point for operationalising such approaches because of its high climate sensitivity and well-established surveillance infrastructure [12].

The review further demonstrates that intersectoral collaboration is strongly endorsed across policies, reflecting growing recognition of One Health and multisectoral approaches. However, governance arrangements for routine climate-health data sharing remain fragmented. The lack of defined mechanisms for routine data exchange between health and meteorological sectors, limited guidance on the use of hydrometeorological data, and absence of standardised analytical frameworks all constrain implementation [33]. Effective EWS require institutionalised collaboration between sectors, interoperable data systems, and clear protocols linking climate signals to health outcomes and decision thresholds [34]. The Vietnamese policy framework provides an enabling foundation but does not yet establish the technical and operational architecture needed for full integration.

Finally, significant implementation challenges persist. Resource limitations, workforce shortages, constrained local capacity, and funding mechanisms oriented towards outbreak response rather than anticipatory action may undermine the sustainability of EWSs. The development of EWS will require new competencies, including climate-health modelling, geospatial analysis, data science, and the interpretation of meteorological information [20]. Without targeted investment in these areas, policy ambitions related to forecasting and digital transformation are unlikely to be fully realised.

Based on these findings, several priority actions emerge. Prioritised recommendations for strengthening climate-informed infectious disease EWSs in Vietnam are presented in Supplementary Table 5.

First, strengthening climate-health governance should be prioritised as a prerequisite for EWS implementation. Establishing formal governance arrangements between health, meteorological, environmental, and other relevant sectors would facilitate systematic access to climate and environmental information for disease risk assessment and forecasting [33,34]. Such arrangements should extend beyond data sharing to include clearly defined roles, accountability mechanisms, interoperability standards, quality assurance procedures, and sustainable financing arrangements [27].

Second, Vietnam would benefit from a dedicated policy and operational framework for infectious disease EWSs. The review found limited guidance on how predictive information should be generated, validated, interpreted, and used in decision-making. A national framework could define the core functions of EWSs, institutional responsibilities, priority diseases, minimum data requirements, model governance arrangements, and mechanisms for integrating forecasting into routine surveillance and preparedness systems. Given their strong climate sensitivity and existing forecasting experience, dengue and other vector-borne diseases may provide appropriate entry points for implementation.

Third, greater attention should be given to strengthening warning-to-action pathways. Consistent with international EWS frameworks, effective warning systems require not only risk detection but also predefined procedures that translate warnings into timely action [4,7]. Developing a tiered warning architecture, supported by clearly defined response measures for different levels of risk, could improve the actionability and legitimacy of early warning information.

Fourth, forecast-based action should be formally incorporated into public health preparedness and financing systems. Establishing authorised forecast-based triggers for activities such as intensified vector control, community engagement, risk communication, stockpiling, or enhanced surveillance could support earlier intervention. Importantly, financing mechanisms should be adapted to permit expenditure based on forecast risk rather than confirmed outbreaks, reducing delays in preventive action.

Fifth, implementation will require investments in workforce development and institutional capacity. Capacity strengthening should target both national technical experts and end-users responsible for interpreting and acting on warnings at provincial and local levels.

Finally, long-term sustainability should be considered from the outset. Stakeholders consistently highlighted concerns that EWSs may remain project-based unless formally embedded within government systems. Clear institutional ownership is therefore required for model maintenance, system operation, validation, warning dissemination, and ongoing technical support. Embedding EWS functions within routine programmes, performance frameworks, and dedicated budget lines will be essential for ensuring sustainability beyond pilot projects and donor-funded initiatives.

The findings of this study should be interpreted in light of several limitations. First, the review focused on national-level policies and regulations and did not examine implementation at provincial or local levels, where practices may vary considerably. Second, the analysis assessed policy content rather than implementation outcomes and therefore may not fully reflect how policies are operationalised in practice. To mitigate this limitation, stakeholder consultations were conducted to provide insights into implementation experiences and governance challenges; however, these perspectives may not capture the full diversity of experiences across regions and sectors. Stakeholder recruitment used purposive sampling and may therefore be subject to selection bias. Participants were drawn from a limited number of provinces and institutions, which may not fully reflect Vietnam’s diverse health system contexts. In addition, participant identification partly relied on professional networks, potentially resulting in uneven representation of perspectives. In addition, although policy documents and interviews were analysed in Vietnamese and translations were reviewed by bilingual researchers, some linguistic nuances and policy-specific meanings may have been lost during translation, introducing potential interpretive bias. Finally, Vietnam’s policy environment is rapidly evolving, and future legislation, implementation guidelines, and technical regulations may further influence the development and operationalisation of infectious disease EWSs.

## Conclusion

Vietnam has made substantial progress in creating a policy environment conducive to infectious disease EWSs. Recent reforms demonstrate growing recognition of climate-sensitive health risks, digital innovation, and multisectoral governance, while the country possesses strong surveillance and outbreak response infrastructure. However, the policy framework has not yet fully transitioned from surveillance-for-response to surveillance-for-anticipation. Key gaps remain in the operational integration of climate information, forecast-based decision-making, warning-to-action mechanisms, data-sharing governance, sustainable financing, and workforce capacity. Within the policies and stakeholder perspectives examined, the major remaining challenges appear to concern operationalisation and institutionalisation rather than recognition of the rationale for EWSs.

## Supplementary Material

Supplementary_Table 3.docx

PRISMA_ScR_Checklist_Policy review.docx

Supplementary_Table 1.docx

Supplementary Table 2.docx

Supplementary_Table 5.docx

COREQ_Checklist_Policy review.pdf

Supplementary_Table 4.docx

## Data Availability

The data that support the findings of this study are available from the corresponding author, C.T.P., upon reasonable request.
