# Supplementary material for: Assessing policy readiness for climate-informed infectious disease early warning systems in Vietnam: a scoping review and stakeholder consultation
Source: Glob Health Action. 2026 Nov 3;19(1):2757958. doi: 10.1080/16549716.2026.2757958 (PMC13757894; doi:10.1080/16549716.2026.2757958)
Supplement: Supplementary file 2 [file ZGHA_A_2757958_SM8706.docx]

# Supplementary Table 4. Policy readiness assessment framework for climate-informed infectious disease EWSs

| **EWS component** | **Assessment criteria** | **Evidence sought** | **Interpretation** | **Example provision** |
| --- | --- | --- | --- | --- |
| **Risk knowledge and assessment** | Policies establish mandates and mechanisms to assess disease risk, climate vulnerability, environmental drivers, and population susceptibility | Requirements for risk assessment, climate adaptation planning, vulnerability mapping, intersectoral risk analysis, use of environmental and climate information | **High readiness:** Explicit mandates, methods, responsibilities, and coordination mechanisms. **Moderate readiness:** General recognition of risk assessment without implementation guidance. **Low readiness:** Limited or no reference to climate-related risk assessment. | National climate adaptation strategies requiring health-sector climate risk assessments |
| **Detection, monitoring and forecasting** | Policies support surveillance, data collection, data integration, analytical capacity, and disease forecasting | Surveillance regulations, electronic reporting systems, data-sharing arrangements, forecasting mandates, model development and validation procedures | **High readiness:** Clear responsibilities, interoperable data systems, forecasting guidance, and validation requirements. **Moderate readiness:** Surveillance established but forecasting functions weakly defined. **Low readiness:** Limited support for forecasting or cross-sector data integration. | Regulations on infectious disease reporting and electronic surveillance systems |
| **Warning dissemination and communication** | Policies establish procedures for issuing warnings, communicating risks, and coordinating alerts across sectors | Alert frameworks, warning thresholds, communication protocols, designated authorities, public risk communication requirements | **High readiness:** Clearly defined warning levels, triggers, authorities, and communication procedures. **Moderate readiness:** Communication mechanisms exist but warning triggers are unclear. **Low readiness:** Limited guidance on warning generation or dissemination. | Risk communication regulations and emergency notification procedures |
| **Preparedness and response capacity** | Policies link warnings to predefined actions, response plans, resource mobilisation, and accountability mechanisms | Response protocols, contingency plans, financing arrangements, workforce requirements, forecast-based action triggers | **High readiness:** Explicit warning-response pathways, financing mechanisms, and implementation responsibilities. **Moderate readiness:** Response systems exist but are not linked to forecasts. **Low readiness:** Limited guidance on anticipatory action or operational response. | Outbreak response regulations and emergency preparedness plans |
| **Cross-cutting governance and institutional arrangements** | Policies establish institutional leadership, intersectoral coordination, accountability, and long-term sustainability | Governance structures, coordinating bodies, data-sharing agreements, legal mandates, workforce development, investment commitments | **High readiness:** Clearly defined institutional roles, coordination mechanisms, and sustainable resource commitments. **Moderate readiness:** Coordination encouraged but mechanisms remain fragmented. **Low readiness:** Unclear leadership, responsibilities, or funding arrangements. | One Health strategies, inter-ministerial coordination mechanisms, digital transformation policies |
