# Supplementary material for: Assessing policy readiness for climate-informed infectious disease early warning systems in Vietnam: a scoping review and stakeholder consultation
Source: Glob Health Action. 2026 Nov 3;19(1):2757958. doi: 10.1080/16549716.2026.2757958 (PMC13757894; doi:10.1080/16549716.2026.2757958)
Supplement: Supplementary file 3 [file ZGHA_A_2757958_SM8708.docx]

# Supplementary Table 5. Prioritised recommendations for strengthening climate-informed infectious disease EWSs in Vietnam

| **Timeframe** | **Recommendation** | **Lead agency** | **Supporting agencies** | **Rationale** |
| --- | --- | --- | --- | --- |
| **Short-term (1-2 years)** | Establish formal data-sharing agreements and interoperability mechanisms between health, meteorological, environmental and related sectors | MOH | VNMHA, NIHE, Pasteur Institutes, Regional Institutes, Provincial CDCs | Improve access to climate and health data required for forecasting and risk assessment |
| **Short-term (1-2 years)** | Develop national warning protocols, including alert levels, trigger thresholds, communication pathways, and predefined response actions | MOH | Provincial Departments of Health, Provincial CDCs, VNMHA | Strengthen the warning-to-action pathway and improve consistency of responses |
| **Medium-term (3-5 years)** | Develop and implement national frameworks for forecasting model validation, evaluation, and operational adoption | MOH | NIHE, Research institutes, Universities, VNMHA | Increase confidence in forecasts and support routine operational use |
| **Medium-term (3-5 years)** | Strengthen workforce capacity in climate-health analytics, forecasting, data science, and risk communication | MOH | Universities, Research institutes, Provincial CDCs | Build the technical expertise required to operate and sustain climate-informed EWSs |
| **Long-term (>5 years)** | Institutionalise climate-informed EWSs within routine public health planning, surveillance, and emergency preparedness systems | MOH | VNMHA, Provincial Authorities, Research institutes | Ensure long-term sustainability and integration into public health decision-making |
| **Long-term (>5 years)** | Establish sustainable financing and forecast-based funding mechanisms to support anticipatory action | MOF and MOH | Provincial Authorities, Development Partners | Enable timely implementation of preventive measures based on forecasted risk |
