# Supplementary material for: Assessing policy readiness for climate-informed infectious disease early warning systems in Vietnam: a scoping review and stakeholder consultation
Source: Glob Health Action. 2026 Nov 3;19(1):2757958. doi: 10.1080/16549716.2026.2757958 (PMC13757894; doi:10.1080/16549716.2026.2757958)
Supplement: Supplementary file 4 [file ZGHA_A_2757958_SM8709.docx]

# Supplementary Table 2: Characteristics of Key Informants Participating in the Study

| Stakeholder Group/Code | Position | Organisation | Main Responsibility | Gender | Age | Years of Experience |
| --- | --- | --- | --- | --- | --- | --- |
| Policymaker 01 | Head, Infectious Disease Control Division | Department of Disease Prevention, Ministry of Health | Responsible for national infectious disease control governance. Provides policy advice on laws, regulations, circulars, and national guidelines; oversees national infectious disease surveillance through reporting systems; and guides provincial health authorities and regional institutes on infectious disease surveillance, prevention, and control activities. | Male | 40–50 | >20 years |
| Policymaker 02 | Deputy Director | Pasteur Institute Nha Trang | Responsible for disease surveillance and prevention in the central coastal region. Oversees surveillance systems, laboratory diagnostics, outbreak forecasting, and infectious disease early warning activities. Serves as a key coordinator within Vietnam’s regional infectious disease surveillance network. | Male | 30–40 | >10 years |
| Policymaker 03 | Vice Head, Communicable Disease Control | National institute of Hygiene and Epidemiology | Provides technical leadership for national epidemiological surveillance, outbreak response, disease modelling, and implementation of infectious disease reporting and warning systems. | Male | 40–50 | >20 years |
| Policymaker 04 | Deputy Director | Agricultural Meteorology Center – Institute of Meteorology, Hydrology and Climate Change | Provides meteorological data, climate modelling, and weather–disease analyses to support development of climate-informed early warning systems for infectious diseases. | Male | 40–50 | >20 years |
| Industry 01 | Head, Digital Health Solutions Division | Viettel Solutions – Viettel Enterprise Solutions Corporation | Leads the development and implementation of digital health technologies and information systems. Supports digital transformation initiatives and develops digital health applications for disease prevention, healthcare delivery, and public health management. | Male | 40–50 | >15 years |
| Practitioner 01 | Deputy Head, Medical Services Department | Tây Ninh Department of Health | Responsible for preventive medicine and communicable disease management at the provincial level. Provides technical and regulatory advice on disease prevention, healthcare services, and infectious disease response; develops provincial preparedness and outbreak response plans. | Female | 40–50 | 23 years |
| Practitioner 02 | Deputy Head, Medical Services Department | An Giang Department of Health | Responsible for the management and coordination of infectious disease prevention and control activities at the provincial level. Advises on preventive medicine, healthcare services, and outbreak response; develops strategies and operational plans for disease prevention and emergency preparedness. | Male | 30–40 | 3 years |
| Technical Officer 01 | Entomologist | Department of Medical Entomology and Zoology, Pasteur Institute in Ho Chi Minh City | Conducts entomological surveillance and vector investigations; trains local health staff on vector control; performs infectious disease virus detection and serotyping in mosquitoes; evaluates vector control interventions; and provides technical guidance on insecticide spraying and outbreak investigations. | Male | 30–40 | 13 years |
| Technical Officer 02 | Epidemiologist | Department of Disease Control and Prevention, Pasteur Institute in Ho Chi Minh City | Conducts surveillance and outbreak investigations for infectious and emerging diseases, including infectious disease. Supports disease prevention and control activities, delivers epidemiological training, and strengthens field epidemiology and preventive medicine capacity among local health workers. | Male | 30–40 | >15 years |
| Technical Officer 03 | Medical Doctor | Department of Disease Control and Prevention, Pasteur Institute in Ho Chi Minh City | Supports infectious disease surveillance and outbreak response, with a focus on infectious disease prevention and control. Conducts epidemiological investigations, trains frontline health workers in early case detection and reporting, applies data analytics and spatial mapping techniques, contributes to outbreak prediction model development, and evaluates early warning systems in urban settings. | Male | 20–30 | >10 years |
| Researcher 01 | Epidemiologist and Professor of Public Health | Faculty of Public Health, University of Medicine and Pharmacy at Ho Chi Minh City | Provides technical expertise in infectious disease surveillance, epidemiological modelling, and disease prevention in the Mekong Delta region. Contributes to workforce capacity building, research, and technology transfer related to surveillance and early warning systems. | Male | >60 | 5 years |
| Researcher 02 | Project Coordinator | DART Project, Oxford University Clinical Research Unit (OUCRU) | Coordinates activities under the DART project, including the development of dengue modelling and early warning tools for Ho Chi Minh City, and conducts implementation research on infectious disease early warning systems. | Male | 20–30 | 3 years |
