# Supplementary material for: Assessing policy readiness for climate-informed infectious disease early warning systems in Vietnam: a scoping review and stakeholder consultation
Source: Glob Health Action. 2026 Nov 3;19(1):2757958. doi: 10.1080/16549716.2026.2757958 (PMC13757894; doi:10.1080/16549716.2026.2757958)
Supplement: Supplementary file 5 [file ZGHA_A_2757958_SM8710.docx]

# Supplementary Table 1: Search term and Search strategy

## Search platform:

Thư Viện Pháp Luật: <https://thuvienphapluat.vn/>

Vietnam National Legal Portal: <https://vietnamlaw.gov.vn/>

Vietnam Ministry of Health: <https://moh.gov.vn/>

Ministry of Agriculture and Environment: <https://mae.gov.vn/>

Vietnam Government Portal: <https://chinhphu.vn/>

## Search date:

From 10 May – 10 July 2026

## Vietnamese keywords:

| Concept | Vietnamese search terms |
| --- | --- |
| Early warning/Early warning system/ Prediction  AND | cảnh báo sớm OR dự báo OR cảnh báo sớm OR hệ thống cảnh báo sớm |
| Infectious disease/Disease outbreak  AND | bệnh truyền nhiễm OR dịch bệnh OR dịch bệnh truyền nhiễm |
| Climate/ Weather | khí hậu OR thời tiết OR khí tượng thuỷ văn |

## Search strings:

Governments’ policies and programmes were searched on legal database using the following search terms:

(“cảnh báo sớm” OR “dự báo” OR “cảnh báo sớm” OR “hệ thống cảnh báo sớm” ) AND (“bệnh truyền nhiễm” OR “dịch bệnh” OR “dịch bệnh truyền nhiễm”) AND (“khí hậu” OR “thời tiết” OR “khí tượng thuỷ văn”)

Number retrieved and Number retained:

Expert-nominated documents were independently verified against inclusion criteria before inclusion
