# Supplementary material for: Assessing policy readiness for climate-informed infectious disease early warning systems in Vietnam: a scoping review and stakeholder consultation
Source: Glob Health Action. 2026 Nov 3;19(1):2757958. doi: 10.1080/16549716.2026.2757958 (PMC13757894; doi:10.1080/16549716.2026.2757958)
Supplement: Supplementary file 7 [file ZGHA_A_2757958_SM8712.docx]

# Supplementary Table 3: Analytical integration matrix

| **Theme** | **EWS domain** | **Policy evidence** | **Stakeholder evidence** | **Illustrative interview codes** | **Convergence between policy and stakeholder evidence** | **Divergence between policy and stakeholder evidence** | **Negative/discrepant cases** |
| --- | --- | --- | --- | --- | --- | --- | --- |
| **Strong foundations for surveillance and outbreak detection** | Detection, monitoring and forecasting; Preparedness and response | Law on Prevention and Control of Infectious Diseases; Circular 54/2015/TT-BYT; Circular 15/2026/TT-BYT | MOH, NIHE, Provincial CDCs | Electronic reporting; Event-based surveillance; Rapid outbreak detection | Both policy documents and stakeholders described well-established surveillance and reporting systems | National stakeholders emphasised system strengths, while provincial stakeholders highlighted operational constraints and resource limitations | Some provincial participants reported inconsistent implementation across local settings |
| **Growing recognition of climate-informed EWSs in national policy** | Risk knowledge and assessment; Detection, monitoring and forecasting | National climate adaptation strategies; Digital transformation policies; Health sector climate adaptation plans | MOH, Meteorological Institute, Universities | Climate adaptation; Forecasting; Climate-health integration | Both sources recognised increasing policy attention to climate-informed approaches | Stakeholders noted that policy recognition has not yet translated into operational guidance | A small number of respondents questioned whether climate-informed EWSs should be prioritised over strengthening routine surveillance |
| **Fragmented governance and data integration arrangements** | Detection, monitoring and forecasting | Data-sharing provisions across health and meteorological sectors; Digital transformation policies | MOH, NIHE, Meteorological Institute, Provincial CDCs | Data sharing barriers; Interoperability; Data ownership | Both sources identified the need for cross-sector data integration | Some respondents considered existing data-sharing arrangements adequate for current operational needs, whereas policies implied broader integration ambitions | A few stakeholders reported successful informal collaboration mechanisms despite perceived policy gaps |
| **Limited operational guidance for forecast generation and adoption** | Detection, monitoring and forecasting | Technical decisions and strategic plans referencing forecasting and AI applications | MOH, Research institutes, Universities, Industry | Model validation; Forecast uncertainty; Scientific evidence | Policy and stakeholder findings both identified the absence of clear validation and adoption frameworks | Policies promote innovation and forecasting, while stakeholders expressed concerns about forecast accuracy and accountability | Some technical experts viewed existing predictive models as sufficiently mature for operational testing |
| **Weak warning-to-action pathways** | Warning dissemination and communication; Preparedness and response | Emergency response regulations; Risk communication guidance | MOH, Provincial CDCs, Departments of Health | Alert thresholds; Trigger mechanisms; Decision-making authority | Both sources highlighted the absence of clear links between warnings and predefined actions | Policies support communication but provide little operational guidance on forecast-based action | Some stakeholders argued that existing outbreak-response protocols could be adapted for forecast-based response |
| **Resource and institutional capacity constraints** | Preparedness and response capacity | Workforce development plans; Public health investment policies; Climate adaptation plans | MOH, Provincial CDCs, Universities | Workforce capacity; Financing; Sustainability | Both policy documents and stakeholders identified workforce and financing needs | Policies often acknowledge capacity development, but stakeholders reported insufficient resources for implementation | A few national respondents considered current institutional capacity adequate if additional guidance and coordination mechanisms were introduced |
